# Targeting the Proteasome in Advanced Renal Cell Carcinoma: Complexity and Limitations of Patient-Individualized Preclinical Drug Discovery

**DOI:** 10.3390/biomedicines9060627

**Published:** 2021-05-31

**Authors:** Jielin Li, Laura Pohl, Julia Schüler, Nina Korzeniewski, Philipp Reimold, Adam Kaczorowski, Weibin Hou, Stefanie Zschäbitz, Cathleen Nientiedt, Dirk Jäger, Markus Hohenfellner, Anette Duensing, Stefan Duensing

**Affiliations:** 1 Molecular Urooncology, Department of Urology, University Hospital Heidelberg, Im Neuenheimer Feld 517, D-69120 Heidelberg, Germany; jhxl2016@outlook.com (J.L.); laura.pohl@stud.uni-heidelberg.de (L.P.); nkorzeni10@gmail.com (N.K.); adam.kaczorowski@med.uni-heidelberg.de (A.K.); houwb@ymail.com (W.H.); 2 Charles River Laboratories, Am Flughafen 12, D-79108 Freiburg, Germany; Julia.Schueler@crl.com; 3 Department of Urology, National Center for Tumor Diseases (NCT), University Hospital Heidelberg, Im Neuenheimer Feld 420, D-69120 Heidelberg, Germany; philipp.reimold@med.uni-heidelberg.de (P.R.); markus.hohenfellner@med.uni-heidelberg.de (M.H.); 4 Department of Medical Oncology, National Center for Tumor Diseases (NCT), Heidelberg University Hospital, Im Neuenheimer Feld 460, D-69120 Heidelberg, Germany; Stefanie.zschaebitz@med.uni-heidelberg.de (S.Z.); Cathleen.nientiedt@med.uni-heidelberg.de (C.N.); dirk.jaeger@med.uni-heidelberg.de (D.J.); 5 Precision Oncology of Urological Malignancies, Department of Urology, University Hospital Heidelberg, Im Neuenheimer Feld 517, D-69120 Heidelberg, Germany; anette.duensing@med.uni-heidelberg.de; 6 Cancer Therapeutics Program, UPMC Hillman Cancer Center, 5117 Centre Avenue, Pittsburgh, PA 15213, USA; 7 Department of Pathology, University of Pittsburgh School of Medicine, 200 Lothrop Street, Pittsburgh, PA 15213, USA

**Keywords:** renal cell carcinoma, drug screening, proteasome inhibitor, carfilzomib

## Abstract

Background: Systemic treatment options for metastatic renal cell carcinoma (RCC) have significantly expanded in recent years. However, patients refractory to tyrosine kinase and immune checkpoint inhibitors still have limited treatment options and patient-individualized approaches are largely missing. Patients and Methods: In vitro drug screening of tumor-derived short-term cultures obtained from seven patients with clear cell RCC was performed. For one patient, a patient-derived xenograft (PDX) mouse model was established for in vivo validation experiments. Drug effects were further investigated in established RCC cell lines. Results: The proteasome inhibitor carfilzomib was among the top hits identified in three of four patients in which an in vitro drug screening could be performed successfully. Carfilzomib also showed significant acute and long-term cytotoxicity in established RCC cell lines. The in vivo antitumoral activity of carfilzomib was confirmed in a same-patient PDX model. The cytotoxicity of carfilzomib was found to correlate with the level of accumulation of ubiquitinated proteins. Conclusions: In this proof-of-concept study, we show that patient-individualized in vitro drug screening and preclinical validation is feasible. However, the fact that carfilzomib failed to deliver a clinical benefit in RCC patients in a recent phase II trial unrelated to the present study underscores the complexities and limitations of this strategy.

## 1. Introduction

Metastatic renal cell carcinoma (RCC) has a poor prognosis with 5-year survival rates between 8% and 12% [1,2]. The therapeutic landscape in metastatic RCC has changed considerably over recent decades and immune checkpoint inhibitors, alone or in combination with VEGF targeting agents, are now accepted first-line therapies [3,4,5]. Despite the introduction of novel drugs and drug combinations, individualized treatment approaches for metastatic RCC are still largely missing, but urgently needed for patients who fail to respond to VEGF targeting drugs and immune oncological agents.

Advanced RCC is characterized by a high degree of genomic and functional intratumoral heterogeneity [6,7]. Under selection pressure, such as systemic anti-cancer therapy, tumor heterogeneity may promote clonal evolution and, ultimately, the outgrowth of treatment-resistant tumor cell populations [8]. This would argue for repeated molecular analyses over the course of systemic treatment and subsequent therapy adjustments according to arising subclonal driver alterations. We and others have shown that such strategies are feasible and can help to discover novel therapeutic vulnerabilities [9,10]. Some RCCs have recently been reported to harbor multiple driver mutations [11]. Whether and how these drivers interact to shape the drug sensitivity of a specific tumor is currently poorly understood. Patient-individualized tumor models and drug screening may help to overcome these uncertainties. This strategy can include cell culture models directly derived from the patient’s tumor tissue, such as short-term cultures or organoids, as well as patient-derived xenograft (PDX) models [12]. A number of studies have shown the feasibility of these approaches [13,14]. Whether these models reflect the genomic heterogeneity and the clonal composition of a tumor is an open question; however, there is evidence to suggest that this may be the case, at least to a certain degree [15,16]. While it is conceivable that the growth conditions in vitro or in a PDX model may favor more aggressive tumor subclones [17], these subclones may ultimately also determine the clinical course of disease.

In the present proof-of-concept study, we demonstrate the feasibility of simultaneous patient-individualized in vitro drug screening and PDX development in metastatic RCC. The potent proteasome inhibitor carfilzomib was identified as the most effective compound in vitro and its antitumoral activity was confirmed in vivo. In light of a negative phase II study of carfilzomib in patients with advanced RCC, published recently [18], complexities and limitations of patient-individualized drug discovery approaches are discussed.

## 2. Materials and Methods

### 2.1. Short-Term Tumor Cell Cultures

Short-term tumor cell cultures were established as previously described in [19]. Briefly, fresh tumor tissue was obtained intra-operatively and transferred to the cell culture facility. Tissue specimens were mechanically minced and incubated in an enzyme cocktail consisting of 40 U/mL collagenase (Sigma-Aldrich, St. Louis, MO, USA), 125 U/mL DNAse (Sigma-Aldrich), and 100 U/mL hyaluronidase (Sigma-Aldrich) at 37 °C for 45 min. Cells were passed through stainless steel sieves and suspended in RPMI 1640 cell culture media supplemented with 5% fetal bovine serum, 100 U/mL of penicillin, 100 U/mL of streptomycin, and 1% l-glutamine (all from Gibco, Carlsbad, CA, USA). All experimental procedures were approved by the Ethics Committee of the University of Heidelberg School of Medicine (S-453/2010). Written informed consent was obtained from all patients.

### 2.2. In Vitro Drug Screening, Inhibitor Treatment and Long-Term Tumor Cell Growth Assays

Short-term tumor cell cultures were subjected to a medium-throughput functional drug screening procedure using the National Cancer Institute’s (NCI) Developmental Therapeutics Program Approved Oncology Drugs Set IV. A full list of compounds can be found under https://dtp.cancer.gov/organization/dscb/obtaining/available_plates.htm (accessed on 21 November 2013). The compounds of the library included chemotherapeutic agents, small molecule inhibitors, and antihormonal substances. Briefly, tumor cells were seeded into 96-well plates with 10,000 cells per well and allowed to adhere overnight. Drugs were then added to the tumor cells at a final concentration of 1 and 10 μM, respectively. The drug concentrations used for hit discovery, i.e., 1 and 10 μM, were based on the published literature [20] and allow comparability between a relatively large variety of compounds with very distinct modes of action. The higher concentration (10 μM) was included based on the known fact that drug resistance is widespread in RCC. No lower concentrations were tested in the initial screening in order to not miss any potentially active compounds. The plates were incubated at 37 °C for 72 h, and cell viability was measured using 3-(4,5-dimethylthiazol-2-yl)-2,5-diphenyltratrazolium bromide (MTT) assays (Thermo Fisher Scientific, Waltham, MA, USA). All experiments were performed in triplicate and the fold-changes over DMSO-treated tumor cells used as controls were calculated. Sorafenib, sunitinib, axitinib and carfilzomib (all Selleck Chemicals, Houston, TX, USA) were used at the concentrations and time intervals indicated. The colony growth assays were performed as previously described in [21]. Established RCC cell lines 786-0, ACHN, A-498, Caki-1, Caki-2 as well as non-cancerous HEK293 cells were obtained from CLS Cell Lines Service (Eppelheim, Germany) and maintained as recommended by the distributor. RCC4 cells were obtained from Sigma-Aldrich. Cell lines were chosen to reflect the heterogeneity of the disease and the *VHL* status [22]. All cell lines were cultured in media supplemented with 10% fetal bovine serum, 50 U/mL penicillin/streptomycin, and 0.5 μg/mL amphotericin B at 37 °C with 5% CO_2_.

### 2.3. PDX Models and In Vivo Drug Testing

Primary tumor cells were injected subcutaneously into the flanks of Naval Medical Research Institute (NMRI)-nude mutant mice. The growth of palpable tumors was monitored and followed by randomized treatment with the pharmacologic agents (vehicle control or 3 mg/kg/d carfilzomib i.v.). The treatment group consisted of five animals; the control group consisted of four animals. To determine therapeutic efficacy, tumors were measured twice weekly. The study was performed in accordance with the recommendations of the Guide for the Care and Use of Laboratory Animals of the Society of Laboratory Animals (GV SOLAS). All animal experiments were approved by the Committee on the Ethics of Animal Experiments of the Freiburg regional council (Regierungspräsidium Freiburg, Abt. Landwirtschaft, Ländlicher Raum, Veterinär- und Lebensmittelwesen—Ref. 35. Permit no.: G-09/58) and performed at Charles River Laboratories, Freiburg, Germany.

### 2.4. Analysis of Ubiquitinated Proteins

The Proteome Profiler™ Array for human ubiquitinated proteins was used as recommended by the manufacturer (R&D Systems, Minneapolis, MN, USA).

### 2.5. Statistical Analysis

Student’s two-tailed *t*-test for independent samples was used wherever applicable. *p* values ≤ 0.05 were considered statistically significant.

## 3. Results

### 3.1. Carfilzomib Has Potent Antitumoral Activities in RCC Cells

**Figure 1:**
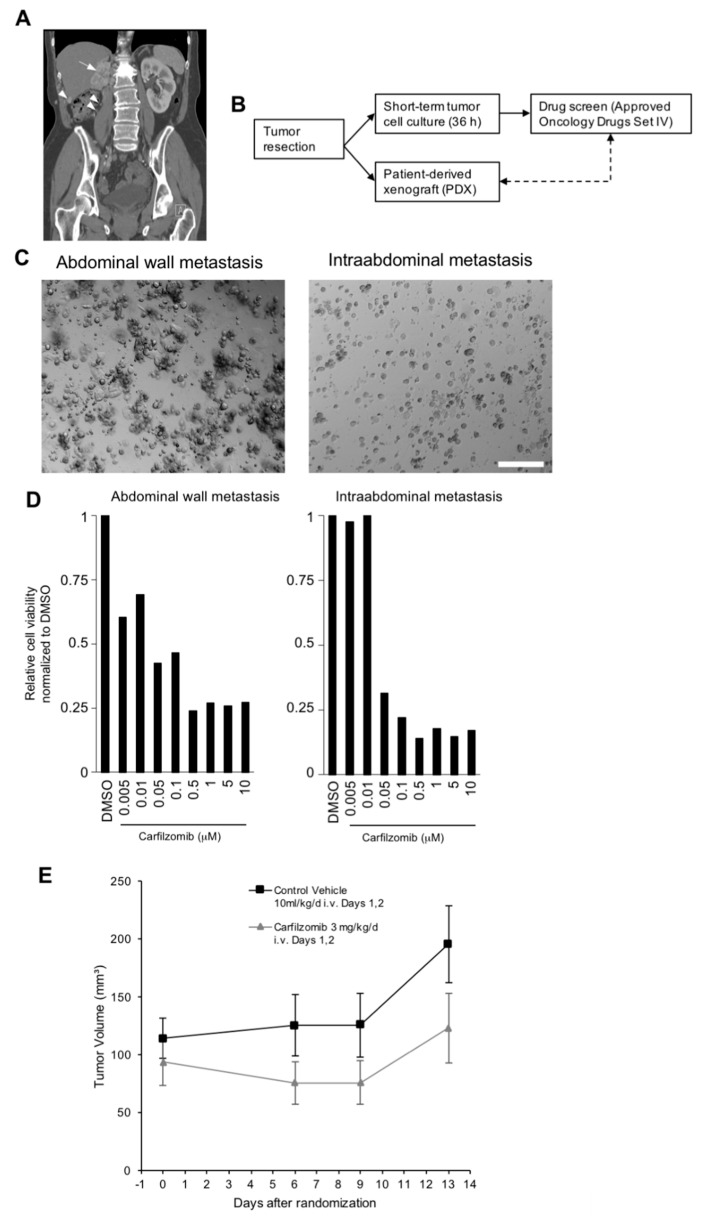
Antitumoral activity of carfilzomib in vitro and in a corresponding PDX model. (**A**) Computed tomography image of a local RCC recurrence (arrow) with examples of metastatic dissemination (arrowheads; patient RCC001). (**B**) Workflow of in vitro drug screening and PDX development. (**C**) Photomicrographs of short-term tumor cell cultures obtained from patient RCC001. Scale bar = 250 μm. Note that tumor cells in the left image are heavily clustered, and thus appear bigger than the cells in the right image. (**D**) Quantification of cell viability of short-term cultures obtained from patient RCC001 after treatment with increasing concentrations of carfilzomib for 72 h. Fold-changes after normalization to control (DMSO) from a single validation experiment are shown. For technical reasons, i.e., a drop in tumor cell proliferation after passage three, we were not able to repeat the experiment. (**E**) Mean tumor volumes (±standard error) in a same-patient PDX model of patient RC001 after treatment with carfilzomib or vehicle control as indicated.

Short-term tumor cell cultures from seven RCC patients were established and used for functional drug screening with the NCI Approved Oncology Drugs Set IV (Table 1). For patient RCC001, two tumor specimens (an abdominal wall metastasis and an intraabdominal metastasis, Figure 1a) were obtained and used for short term culture and drug testing as well as establishment of a PDX model (from the intraabdominal metastasis) for later use for in vivo validation experiments (Figure 1b,c).

A drug hit was defined as a ≥0.5-fold change in cell viability by a compound (i.e., IC_50_ was reached), and only drug screens with at least one compound producing such a reduction of cell viability were considered successful in our analyses [23]. Following this definition, successful drug screening results were obtained from four of seven patients (Table 1).

The proteasome inhibitor carfilzomib was a hit in three of the four successful drug screens, with reduction in tumor cell viability ranging from a 0.19-fold to a 0.28-fold change (RCC001, RCC008, and RCC024; Table 1). Other hits were the related proteasome inhibitor bortezomib (0.25-fold change in RCC008), the transcriptional inhibitor dactinomycin (0.24-fold change in RCC001), the DNA methyltransferase inhibitor decitabine (0.16-fold change in RCC024), the BRAF inhibitor dabrafenib (0.24-fold change in RCC008), the hormonal alkylating agent estramustine (0.24-fold change in RCC024) and a number of tyrosine kinase inhibitors (erlotinib, nilotinib, ponatinib; Table 1). Throughout the drug screens, the vast majority of drugs showed only minor effects on cell viability at both concentrations, 1 and 10 μM, which indicates resistance of the tumor cells to a wide spectrum of antineoplastic substances.

We were able to further validate the result that carfilzomib is highly effective in RCC001 by testing short-term tumor cell cultures obtained from two distinct anatomic locations (Figure 1c,d). Both short-term cultures showed a ≥50% reduction of cell viability after carfilzomib treatment starting at drug concentrations of 0.005 and 0.05 μM, respectively (Figure 1d). Furthermore, an in vivo validation of the antitumoral effects of carfilzomib was performed using a PDX model from the same patient. While the in vitro results indicate a cytotoxic effect, carfilzomib had mainly a cytostatic effect in vivo with suppression of tumor growth when compared to vehicle control (Figure 1e). The carfilzomib experiment was continued until day 50 after randomization. After day 30, the tumors of the treatment group were invariably smaller than in the control group with a maximal difference at day 41 (mean absolute tumor volume 569.5 mm^3^ in the control group versus 374.6 mm^3^ in the carfilzomib group).

Taken together, functional drug screening of tumor-derived short-term cultures and validation in a same-patient PDX model demonstrate antitumoral effects of the proteasome inhibitor carfilzomib in advanced RCC.

### 3.2. Antitumoral Activity of Carfilzomib in Established RCC Cell Lines

**Figure 2:**
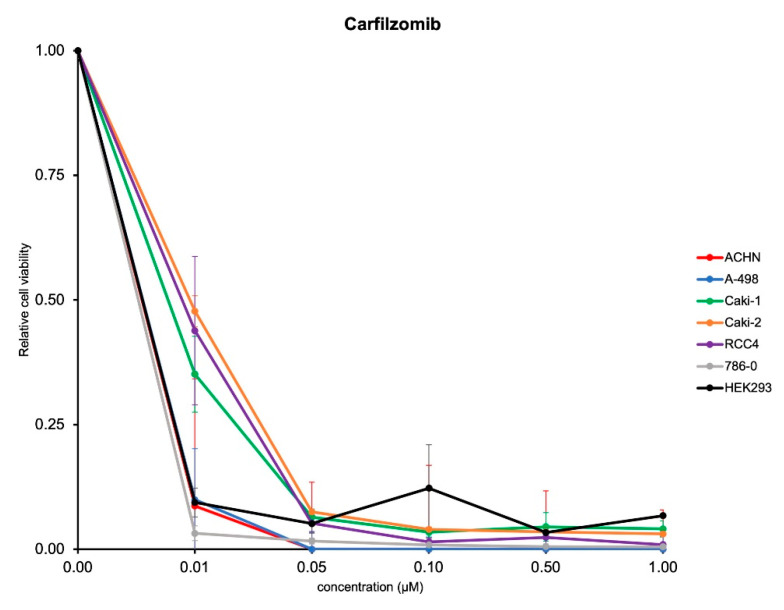
Carfilzomib exerts antitumoral activity in established RCC cell lines and HEK293 cells. Quantification of cell viability of established cell lines after treatment with carfilzomib for 72 h at the concentrations indicated. Results from three independent experiments (mean ± standard error) are shown after normalization to control (DMSO).

We next investigated whether carfilzomib has acute antitumoral activity in established RCC cell lines as well as non-cancerous, kidney-derived HEK293 cells. First, we tested the response to carfilzomib in these cell lines and found growth-inhibitory activities in all cell lines, starting at a concentration of 0.01 μM (Figure 2).

**Figure 3:**
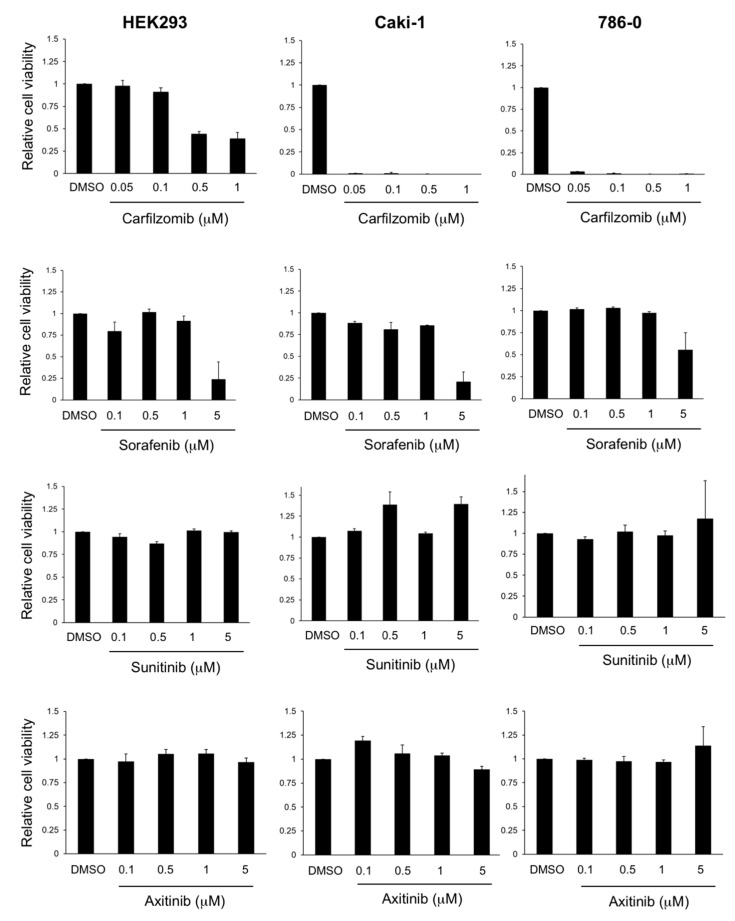
Carfilzomib shows superior in vitro antitumoral activity compared to TKIs. Quantification of cell viability of HEK293, Caki-1 and 786-0 cell lines after treatment with carfilzomib or TKIs at the concentrations indicated for 72 h. Results from three independent experiments (mean ± standard error) are shown after normalization to control (DMSO).

Next, we performed a more detailed analysis using Caki-1 cells (*VHL* wildtype) and 786-0 cells (*VHL* mutated), with HEK293 cells used as controls, and analyzed the response to carfilzomib in comparison to sunitinib, sorafenib and axitinib, which are VEGF receptor targeting receptor tyrosine kinase inhibitors (TKIs) approved for the treatment of metastatic RCC (Figure 3). First, we confirmed the acute cytotoxicity of carfilzomib in Caki-1 and 786-0 cells, which was found to be less pronounced in HEK293 cells. In contrast, sunitinib and axitinib did not induce cytotoxic effects in all three cell lines used, which is in line with their mode of action to mainly target endothelial cells [24]. Sorafenib showed moderate cytotoxic activity but only at the highest drug concentration of 5 μM, suggesting off-target effects (Figure 3).

**Figure 4:**
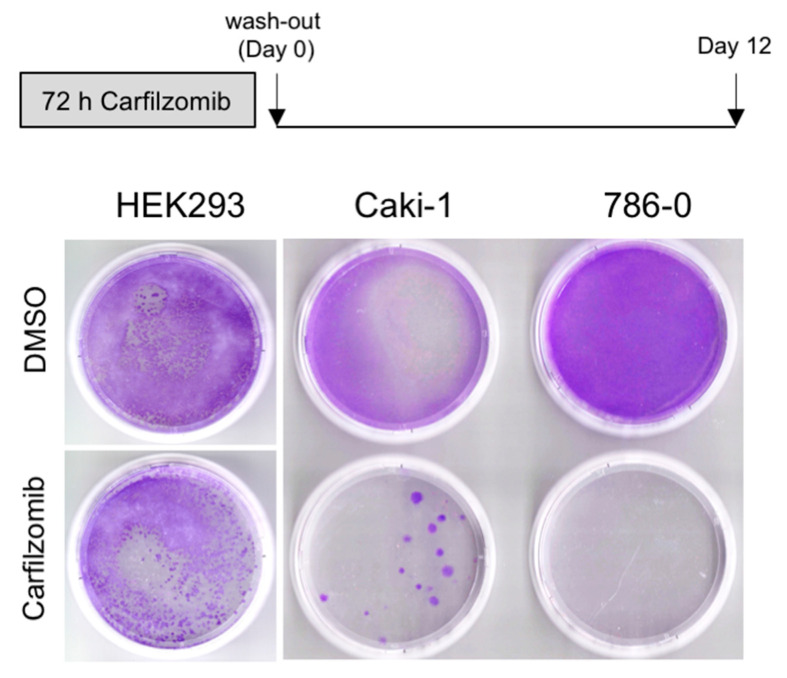
Long-term antitumoral effects of carfilzomib are cell type-dependent. Colony growth assay comparing non-cancerous HEK293 cells to Caki-1 (*VHL* wildtype) and 786-0 (*VHL*-deficient) RCC cell lines over 12 days. Note the absence of colonies in 786-0 cells.

We next sought to explore whether carfilzomib has long-term growth-suppressive effects on RCC cells. Caki-1 and 786-0 cells as well as HEK293 cells were treated for 72 h with 0.1 μM carfilzomib or DMSO, followed by drug wash-out and monitoring of colony growth for 12 days. As shown in Figure 4, there was a highly effective long-term growth suppression by carfilzomib in 786-0 cells and to a lesser extent in Caki-1 cells, while carfilzomib was significantly less effective in non-cancerous HEK293 cells.

Taken together, these results confirm the acute and long-term antineoplastic activity of carfilzomib in RCC cells.

### 3.3. The Level of Accumulation of Ubiquitinated Proteins Correlates with the Antineoplastic Activity of Carfilzomib

**Figure 5:**
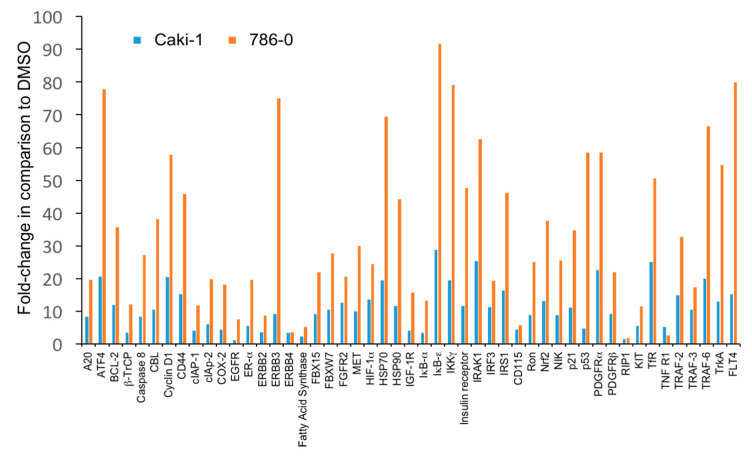
Antitumoral effects of carfilzomib correlate with the level of accumulation of ubiquitinated proteins. Quantification of fold-changes of ubiquitinated proteins in Caki-1 cells and 786-0 cells after treatment with 10 μM carfilzomib or control (0.1% DMSO) for 6 h. Results were normalized to DMSO and corrected for background. Note the higher accumulation of ubiquitinated proteins in 786-0 cells. Results from a representative experiment are shown.

We next investigated the difference in the antitumoral activity of carfilzomib in 786-0 and Caki-1 cells in greater detail. Given the function of carfilzomib as a proteasome inhibitor, we hypothesized that the observed differences may be related to the extent of accumulation of ubiquitinated proteins. Carfilzomib- or DMSO-treated cells were collected and the increase in ubiquitinated proteins was measured using an array for ubiquitinated proteins (Figure 5). Remarkably, carfilzomib induced almost invariably higher levels of ubiquitinated proteins in 786-0 cells in comparison to Caki-1 cells. Ubiquitinated proteins that accumulated over 50-fold in carfilzomib-treated 786-0 cells were ATF4, cyclin D1, ErbB3, FLT4, SP70, IκB-ε, IKKγ, IRAK1, p53, PDGFRα, TfR, TRAF-6, and TrkA (Figure 5).

Taken together, these results suggest that the antitumoral effects of carfilzomib correlate with the level of accumulation of ubiquitinated proteins, suggesting proteotoxic stress as the underlying mechanism.

## 4. Discussion

In this proof-of-concept study, we show that patient-individualized in vitro drug screening and subsequent in vivo validation is feasible and can lead to new candidate drugs for the treatment of advanced RCC. The main hit in our drug screens was the proteasome inhibitor carfilzomib. Carfilzomib showed antitumoral activity not only in short-term tumor cell cultures but also in a same-patient PDX model. In long-term in vitro experiments, carfilzomib was found to be more potent in a RCC cell line with *VHL*-deficiency compared to a cell line harboring wildtype *VHL*. This cell-type-specific antitumoral activity of carfilzomib was found to correlate with the level of accumulation of ubiquitinated proteins, suggesting proteotoxic stress as the underlying mechanism.

Carfilzomib is a potent, second-generation, irreversible inhibitor of the chymotrypsin-like activity of the proteasome [25]. Like the first-in-class proteasome inhibitor, bortezomib, carfilzomib is approved for the treatment of multiple myeloma [26].

Unfortunately, in a recent phase II study of nine patients with treatment-refractory metastatic clear cell RCC, there was an absence of antineoplastic activity of carfilzomib [18]. All nine RCC patients progressed with a median time interval of 1.8 months, and the objective response rate was 0%. The rationale for this small clinical trial was the observation that certain *VHL* mutations lead to a destabilized pVHL with at least partially retained functionality [27]. The *VHL* tumor suppressor gene is inactivated in the majority of clear cell RCCs. pVHL is part of an E3 ubiquitin ligase complex that mediates the ubiquitination of the a subunit of the heterodimeric transcription factor hypoxia-inducible factor (HIF) under normoxic conditions. In case of hypoxia, and when VHL is inactivated, HIFs accumulate and activate signaling pathways that lead, among others, to enhanced angiogenesis via the VEGF/VEGF receptor (VEGFR) axis. This pathway is the basis for established and novel therapeutic interventions in RCC that target VEGF, VEGFR or HIF2α [28].

In a situation, however, where destabilized pVHL with at least partially retained functionality is expressed, pVHL activity could potentially be rescued by inhibition of the proteasome with a subsequent re-activation of HIF degradation [27]. Given that the proteasome inhibitor bortezomib had shown more promising results in patients with advanced RCC [29,30,31], the absence of antineoplastic activity of carfilzomib in the study by Hasanov et al. [18] is perplexing. There are a number of possible explanations for the discrepancy between our preclinical results and the clinical results by Hasanov and colleagues [18].

First, it is conceivable that short-term cultures and established cell lines do not adequately reflect the complex microenvironment in a patient tumor [32].

Second, proteasome inhibition has been reported to attenuate immune cell functions [33]. RCC is a tumor entity in which immune surveillance has long been implicated in the control of malignant growth and progression. The therapeutic efficacy of immune checkpoint inhibitors in metastatic RCC underscores this notion [3]. Despite findings suggesting that tumor-infiltrating cytotoxic T cells are frequently exhausted in RCC [34] further suppression of the immune system could conceivably promote tumor progression in patients with advanced RCC.

There are a number of additional points that need to be taken into account when patient-individualized drug screening and validation are considered. The screening set-up may have favored finding drugs like carfilzomib, since it is known for relatively fast apoptosis induction, while, for example, TKIs are more dependent on cell growth and inhibition of signal transduction pathways. In addition, the target structure of TKIs may not be expressed by the cells analyzed. As such, for drugs with different types of kinetics and mechanisms of action, different assays may be appropriate [35].

It may be also possible to exploit the drug screening results for potential combination therapies. For example, in patient RCC006, carfilzomib and the EGFR-targeting TKI erlotinib were both found as hits (Table 1). A previous study has shown that the combination of carfilzomib and erlotinib has synergistic antitumoral effects both in vivo and in vitro [36]. Carfilzomib exerts cytotoxic effects in the 1 to 0.1 nM range when used as a single agent [36,37]. Through the combination with erlotinib, a carfilzomib concentration that was approximately one order of magnitude lower than when used as a single agent was able to lead to significant cytotoxicity [36]. Hence, combination treatment may trigger synergistic antitumoral effects, thus allowing the use of lower drug concentrations.

The off-label use that almost inevitably follows drug screening efforts has legal ramifications that can prolong or prevent drug procurement. In addition, low engraftment rates and slow tumor growth can delay the generation of PDX models. Moreover, serial passaging has been shown to alter drug sensitivities and evolutionary trajectories away from the primary tumor [17,38,39]. In our study, patient RCC001 did not receive carfilzomib due to rapidly declining performance status.

Further limitations of our proof-of concept study are the small sample size and the lack of genetic information of the tumors. Unfortunately, *VHL* mutation testing has not been performed. However, since the vast majority of patients with clear cell RCC harbor inactivated *VHL*, it is highly likely that this is also the case in our patients. We found a more pronounced accumulation of ubiquitinated proteins in a *VHL*-deficient RCC cell line pointing to a more general role of pVHL in the elimination of abnormal proteins, as previously suggested [40].

Taken together, our results show that patient-individualized drug screening and parallel in vivo validation is feasible but at the same time underscore the complexity of this approach. Further studies are warranted to understand the apparent disconnection between preclinical and clinical findings and to optimize tools for drug discovery in advanced RCC.

## Figures and Tables

**Table 1:** Patient characteristics and in vitro drug screening results.

Patient	Sex/Age at Diagnosis (Years)	RCC Histology	Tumor Stage	Grade	Time to Tumor Progression (Months)	Origin of Specimens Used for Functional Drug Screening	Top Drug Screen Hits (Fold Change in Cell Viability at a 1 μM Concentration Normalized to DMSO)
RCC001	F/58	clear cell	pT2a, cN0, cM0	2	37.6	Abdominal wall metastasis/Intraabdominal metastasis	**Carfilzomib** (0.19) Dactinomycin (0.24)
RCC006	M/48	clear cell	pT3a, pN0 (0/6) pM1 (gall bladder)	3	n/a	Tumor nephrectomy	Dactinomycin (0.67) Erlotinib (0.68) **Carfilzomib** (0.72)
RCC008	M/59	clear cell	pT3, cN1, cM1 (OSS, PUL, BRA, pericardium)	3–4	n/a	Tumor nephrectomy	Dabrafenib (0.24) Bortezomib (0.25) **Carfilzomib** (0.28)
RCC014	M/53	sarcomatoid (major), clear cell (minor)	pT1a, cN0, cM0	4	7.7	Local recurrence	Imatinib (0.62) Capecitabine (0.64) Vorinostat (0.64)
RCC016	M/57	clear cell	pT2, cN0, cM0	2	182.5	Local recurrence/Intraabdominal metastases	Afatenib (0.94)
RCC024	M/67	clear cell	pT3a, cN1, pM1 (OSS, PUL)	2	n/a	Tumor nephrectomy	Decitabine (0.16) Erlotinib (0.17) **Carfilzomib** (0.23) Estramustine (0.24)
RCC025	M/59	clear cell	pT3c, cN0, cM0	unknown	14.3	Peritoneal recurrence	Nilotinib (0.38) Ponatinib (0.43)

Only drug screens with at least one hit, i.e., compound producing a ≥0.5-fold change in tumor cell viability compared to DMSO controls, were considered (grey fields).

## Data Availability

Data are available from the corresponding author upon reasonable request.
